# High-Gain Graphene Transistors with a Thin AlO*x* Top-Gate Oxide

**DOI:** 10.1038/s41598-017-02541-2

**Published:** 2017-05-25

**Authors:** Erica Guerriero, Paolo Pedrinazzi, Aida Mansouri, Omid Habibpour, Michael Winters, Niklas Rorsman, Ashkan Behnam, Enrique A. Carrion, Amaia Pesquera, Alba Centeno, Amaia Zurutuza, Eric Pop, Herbert Zirath, Roman Sordan

**Affiliations:** 1 L-NESS, Department of Physics, Politecnico di Milano, Polo di Como, Via Anzani 42, 22100 Como, Italy; 2 Department of Microtechnology and Nanoscience, Chalmers University of Technology, Gothenburg, 41296 Sweden; 3 Department of Electrical and Computer Engineering, University of Illinois, Urbana, IL 61801 USA; 4 Graphenea, Avenida de Tolosa 76, 20018 Donostia/San Sebastián, Spain; 5 Department of Electrical Engineering, Stanford University, Stanford, CA 94305 USA

## Abstract

The high-frequency performance of transistors is usually assessed by speed and gain figures of merit, such as the maximum oscillation frequency *f*_max_, cutoff frequency *f*_T_, ratio *f*_max_/*f*_T_, forward transmission coefficient *S*_21_, and open-circuit voltage gain *A*_v_. All these figures of merit must be as large as possible for transistors to be useful in practical electronics applications. Here we demonstrate high-performance graphene field-effect transistors (GFETs) with a thin AlO*x* gate dielectric which outperform previous state-of-the-art GFETs: we obtained *f*_max_/*f*_T_ > 3, *A*_v_ > 30 dB, and *S*_21_ = 12.5 dB (at 10 MHz and depending on the transistor geometry) from *S*-parameter measurements. A dc characterization of GFETs in ambient conditions reveals good current saturation and relatively large transconductance ~600 S/m. The realized GFETs offer the prospect of using graphene in a much wider range of electronic applications which require substantial gain.

## Introduction

Graphene is one of the most intensively investigated two-dimensional materials for electronics owing to its large charge carrier mobility (>5,000 cm^2^/(Vs) at room temperature)^1^, which is almost equal between holes and electrons^2^, and large saturation velocity^3^. However, despite intensive research efforts, there are no graphene-based electronic devices available on the market at present. The lack of a bandgap in graphene prevents GFETs from being turned off, which results in large off-state currents and high static power dissipation, and thus represents a fundamental obstacle for the development of graphene-based logic gates^4^.

In contrast, turning off transistors is not required for most analog applications, but the absence of a bandgap weakens the drain current saturation required for high gain operation. Therefore, improving current saturation in GFETs in combination with high mobility of graphene, represent a promising path in the field of high-frequency analog electronics. One possible way to improve current saturation in GFETs is to use ultra-clean samples exhibiting high saturation velocity on substrates with high phonon energy and small density of defects, e.g., h-BN^5^. However, this technology is not yet mature enough to be applied in a large-scale production and is limited by the high cost and complex processing. Similarly, patterning graphene into nanoribbons to open a bandgap reduces the charge carrier mobility and therefore the on-state current, eliminating advantage of improved current saturation. A different approach consists of using thin gate dielectrics in order to assist the channel “pinch-off” and reduce the effect of intrinsic carriers and interfacial traps, which consistently improves saturation^6^. A thinner oxide (thickness ${t}_{{\rm{ox}}}$) also improves the transconductance (*g*_m_) which is proportional to ${t}_{{\rm{ox}}}^{-1}$.

Here we demonstrate high-frequency GFETs on conventional SiO_2_ substrates with improved drain current saturation yielding high-gain operation. The current saturation was improved by utilizing a thin (*t*_ox_ ~ 4 nm) AlO_*x*_ top-gate insulator with good dielectric constant (ε_r,ox_ ~ 6.4). This resulted in improved oxide capacitance (*C*_ox_ = 1.37 *μ*F/cm^2^)^7, 8^ and therefore strong gate control over carriers in the graphene channel. As a consequence, the output conductance (*g*_d_) was reduced well below 50 S/m (normalized by the channel width *W*) leading to very large values of the open-circuit voltage gain *A*_v_ > 30 dB and forward gain *S*_21_ = 12.5 dB (at 10 MHz) in GFETs with the channel widths *W* = 10 *μ*m and *W* = 100 *μ*m, respectively, which are the highest gains measured in GFETs so far^9–12^. This low output conductance also contributed to a large ratio between the extrinsic maximum oscillation frequency (*f*_max_) and cutoff frequency (*f*_T_) of ~3, which is unusually high for GFETs, in which *f*_max_/*f*_T_ typically ranges from <1^13, 14^ to ~1.5^15, 16^ to 3.3^17^. However, the highest measured extrinsic *f*_max_ was 21.3 GHz highlighting the need for alternative substrates with less charge traps^14^ and cleaner graphene transfer process^16^.

GFETs comprising the same type of gate insulator have been successfully implemented in the past in more complex circuits like integrated inverters and ring oscillators capable of performing signal generation up to 4.3 GHz^18^, voltage gain up to 13 or 22.3 dB^19^, mixing and modulation^7^. However, a direct investigation of the high frequency performance of these GFETs has not been performed so far. In this work, different figures of merit, such as *f*_max_, *f*_T_, ${A}_{{\rm{v}}}$ and ${S}_{21}$, were extracted from the $S$-parameter measurements of the individual GFETs to investigate their performance and scaling.

## Experimental

**Figure 1:**
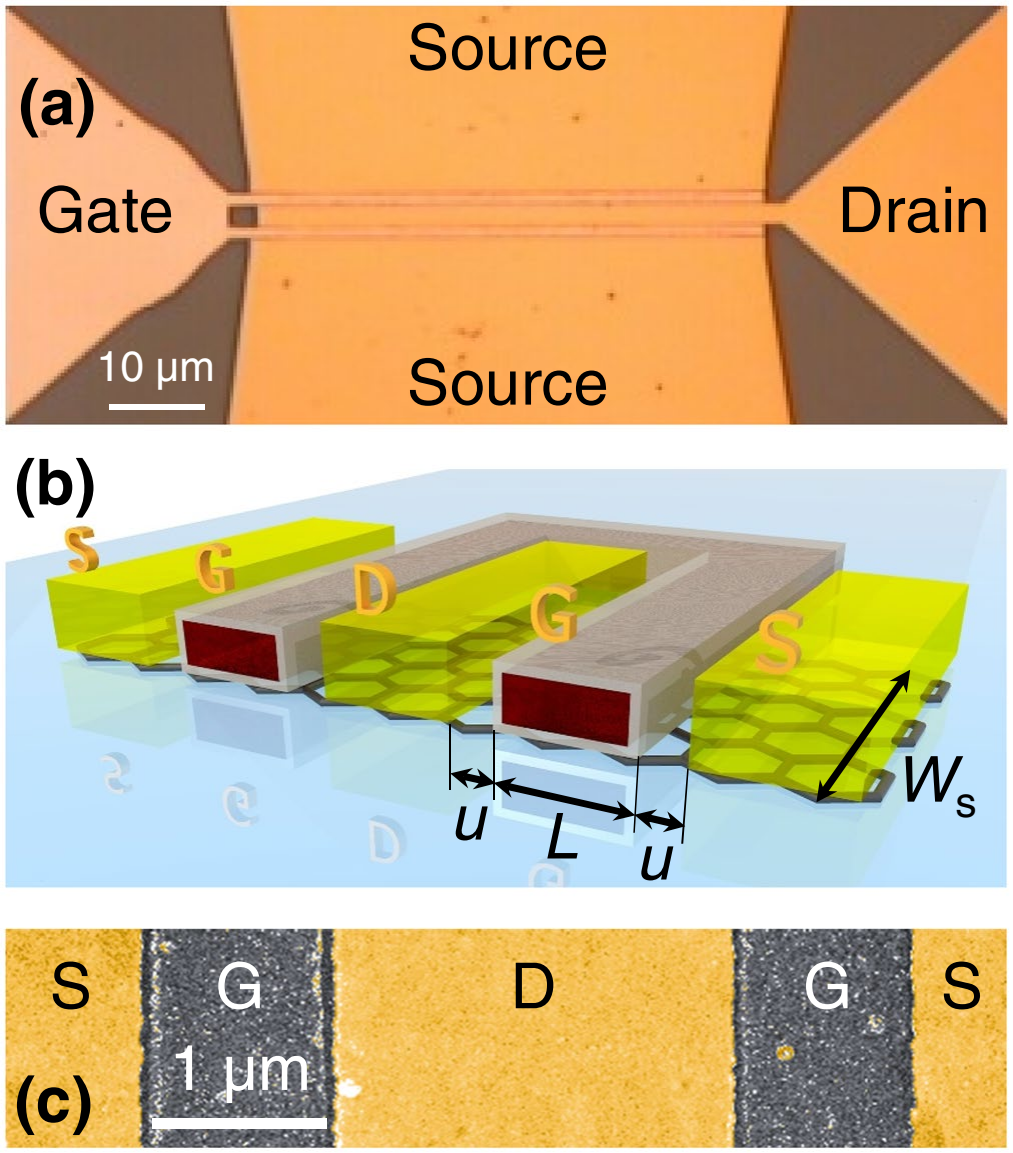
High-frequency GFET. (**a**) Optical image of one of the fabricated GFETs with the gate length *L* = 1.1 *μ*m. Graphene stripe cannot be seen as it is completely covered by the contacts. (**b**) Schematic of the central part of the GFET. Source (S) and drain (D) contacts (Au; yellow) are separated by an underlap (length *u*) from the gate (G) contact (Al; red core), which is covered by an insulating layer (AlOx; gray shell). Width of graphene stripes ${W}_{{\rm{s}}}$ was 5, 20 and 50 *μ*m. All GFETs had the same contact width (~50 *μ*m) regardless of the stripe width. The channel width $W=2{W}_{{\rm{s}}}$. (**c**) Scanning electron microscopy image of the central part of one the fabricated GFETs with the gate length *L* = 1 *μ*m. The underlap is $u=100$ nm.

Monolayer graphene grown by chemical vapor deposition (CVD) on Cu was used in the fabrication of the investigated coplanar GFETs. After the growth, graphene was transferred to high-resistivity (~5 kΩcm) Si substrates with a 1 *μ*m thick top layer of SiO_2_ by a wet process^4^. The graphene stripes were defined by e-beam lithography followed by reactive ion etching in oxygen plasma. The coplanar GFETs were fabricated as depicted in Fig. 1. The channel width *W* was 10, 40 and 100 *μ*m while the gate length *L* was 0.8, 0.9, 1, 1.1 and 2.2 *μ*m. Top gates were patterned by e-beam lithography followed by e-beam evaporation of 100 nm of Al. A thin (${t}_{{\rm{ox}}} \sim 4$ nm) native layer of AlO_*x*_ was formed on all surfaces of the Al gates (including the surface at the interface with graphene) by exposing the samples to air^8, 20–23^. The source/drain contacts were fabricated in the final e-beam lithography step, separated by a very small underlap (*u* *~* 100 nm) from the gate. Such underlaps introduce access resistances between the source/drain and gate which cannot be gated and therefore reduce transconductance and gain. The access resistances can be eliminated by a self-aligned transistor layout^4, 21^ in which the source and drain are overlapped with the gate. However, we found that overlaps introduce additional parasitic capacitances which have more detrimental effect on the high-frequency response of the transistors than access resistances. Typically, self-aligned GFETs with overlapped gate and source/drain exhibited ~3 times smaller *f*_max_ compared to GFETs with (small) underlaps. Such deterioration of the high-frequency response has not been observed in self-aligned T-gate GFETs on flexible substrates^23^. This is probably because the sufficiently large underlaps have been created by shadowing the evaporated source/drain material by the T-gates^23^. The source/drain contacts were made of Au (100 nm thick, without an additional adhesion layer) because we found that pure Au^7^ provides the lowest contact resistance to graphene (~250 Ω*μ*m) among the several tested metal combinations (e.g., Ti/Au, Ni/Au, Pd/Au).

## Results and Discussion

All GFETs were initially characterized at dc to find the optimum biasing conditions for the *S*-parameter characterization. In ambient air, most of the fabricated GFETs exhibited p-type behavior at low biases with the Dirac voltage ${V}_{0} \sim 0.25$ V. A small hysteresis was observed in the dc characteristics of the GFETs. The shift of the Dirac voltage was ~12% of the ${V}_{{\rm{GS}}}$ sweeping range and it did not have any influence on ${g}_{{\rm{m}}}$ and ${g}_{{\rm{d}}}$. We found that the transconductance of the GFETs was larger in the p-type regime (*V*_GS_ < *V*_0_) than in the n-type regime (*V*_GS_ > *V*_0_). This can be attributed to the larger contact resistance in the n-type regime due to the formation of a “pnp” junction between the large workfunction Au contacts and the n-type channel^24, 25^. Moreover, the hole mobility was also found to be slightly larger than the electron mobility, as expected for graphene deposited on SiO_2_ substrates^26, 27^. Therefore, the GFETs were biased in the p-type regime to maximize the transconductance during the *S*-parameter characterization. In order to also minimize the output conductance, the GFETs were measured in the p-type saturation regime (*V*_DS_ < *V*_GS_ − *V*_0_ < 0). *S*-parameter measurements were performed in a Cascade Microtech Summit 12561B probe station. Input and output ac signals were applied by Anritsu Vectorstar MS4647A vector network analyzer. Gate and drain dc biasing was applied by Keithley 2600 source meters. The GFETs were probed by Cascade Microtech 110-A probes with a 100 *μ*m-pitch.

**Figure 2:**
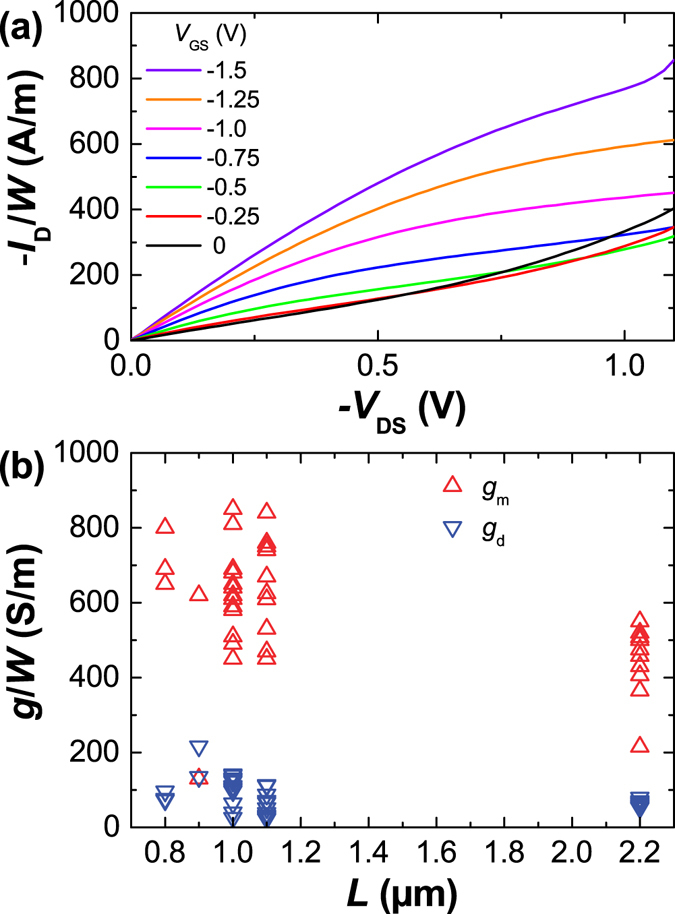
Output characteristics and small-signal conductances of the fabricated GFETs in ambient air. (**a**) Drain current ${I}_{{\rm{D}}}$ as a function of ${V}_{{\rm{DS}}}$ for different ${V}_{{\rm{GS}}}$ in a GFET with $W=10$ *μ*m and $L=1$ *μ*m. The onset of saturation is at ${V}_{{\rm{DS}}}={V}_{{\rm{GS}}}-{V}_{0}$ and it moves to larger $|{V}_{{\rm{D}}S}|$ at larger $|{V}_{{\rm{G}}S}|$. (**b**) The transconductance ${g}_{{\rm{m}}}$ and output conductance ${g}_{{\rm{d}}}$ of the fabricated GFETs at the operating point at which they exhibit the largest voltage gain ${A}_{{\rm{v}}}$.

Figure 2 shows the output characteristics and conductance of a typical GFET with *L* = 1 *μ*m and *W* = 10 *μ*m for several negative values of *V*_GS_, i.e., for transistor operation deep into the p-type regime. Additional advantage of operating p-doped GFETs in the p-type regime is that larger drain currents *I*_*D*_ can be reached compared to the n-type regime. This is because larger values of $|{V}_{{\rm{GS}}}-{V}_{0}|$ for *V*_0_ > 0 can be obtained for negative *V*_GS_ than for positive *V*_GS_ since |*V*_GS_| is limited by the breakdown voltage *V*_b_ of the gate oxide (here *V*_b_ = 2.9 V). At small $|{V}_{{\rm{DS}}}|$, the graphene channel is uniformly p-doped and the GFET is in the ohmic regime exhibited by the linear *I*_D_ − *V*_GS_ curves. A larger $|{V}_{{\rm{DS}}}|$ reduces the hole concentration near the drain leading to charge neutrality and the onset of saturation at ${V}_{{\rm{DS}}}={V}_{{\rm{GS}}}-{V}_{0}$. At even larger $|{V}_{{\rm{DS}}}|$, the charge neutrality point moves towards source with electron accumulation on the drain side. However, as long as $|{V}_{{\rm{DS}}}|$ is not too large, the electron accumulation is not significant and the output conductance approaches zero reflecting strong current saturation. In this regime, the thin dielectric exerts strong capacitive control over the drain current leading to a larger channel depletion rate which improves the drain current saturation^6^. For $|{V}_{{\rm{DS}}}|\gg |{V}_{{\rm{GS}}}-{V}_{0}|$, the current saturation is lost due to significant electron accumulation between the charge neutrality point and drain. This can be observed in the first three plotted curves ($|{V}_{{\rm{GS}}}|\le 0.5$ V) in Fig. 2(a).

**Figure 3:**
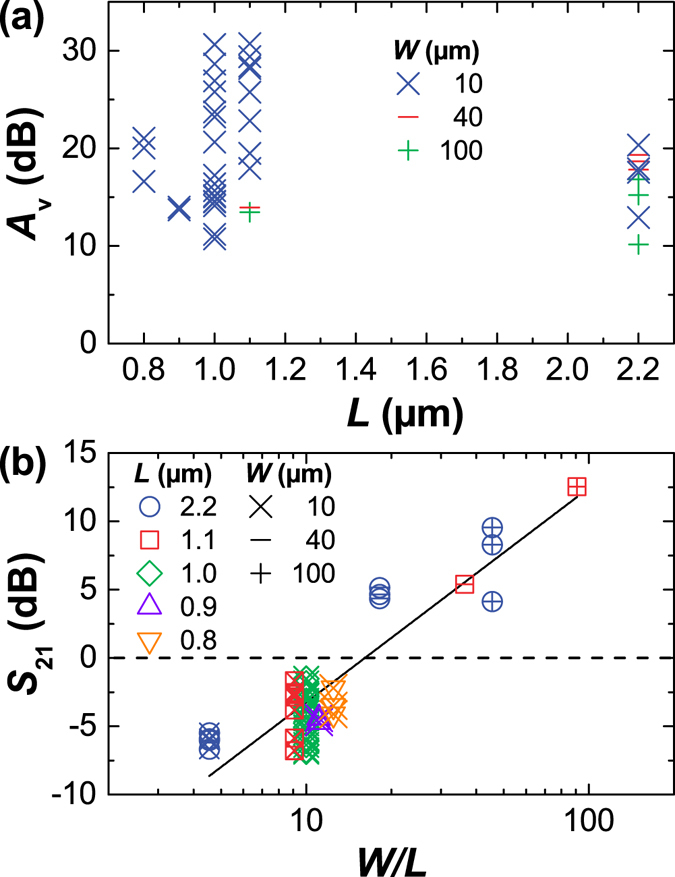
The highest gain in each of the fabricated GFETs at 10 MHz. (**a**) The open circuit voltage gain ${A}_{{\rm{v}}}$ as a function of the gate length *L*. (**b**) The forward gain ${S}_{21}$ as a function of $W/L$. The highest value of 12.5 dB was obtained for $W=100$ *μ*m and $L=1.1$ *μ*m. A $W/L$ fit is suggested by the black line because ${S}_{21}$ scales with ${g}_{{\rm{m}}}$ and therefore with $W/L$.

The highest values of the open-circuit voltage gain ${A}_{{\rm{v}}}=|{Z}_{21}|/|{Z}_{11}|$ measured for each of the fabricated GFETs are shown in Fig. 3(a). The gain was extracted from the *S*-parameter measurements of the GFETs biased at negative *V*_DS_ and *V*_GS_. The open-circuit voltage gain is equal to the intrinsic transistor gain $A={g}_{{\rm{m}}}/{g}_{{\rm{d}}}$ at low frequencies. The intrinsic gain *A* is not expected to scale with *W* or *L* because *g*_m_ scales with *W*/*L* while *g*_d_ approximately scales with *W/L*. However, due to the influence of the contact resistance, the intrinsic gain *A* and consequently *A*_v_ were smaller at shorter *L*. The largest measured *A*_v_ was above 30 dB at 10 MHz for GFETs with $L \sim 1$ *μ*m and *W* = 10 *μ*m. This is the highest voltage gain reported for GFETs so far^9–11^. The notable scatter of gain values can be attributed to device-to-device variability from the intrinsic non-uniformity of CVD graphene and contact misalignment. Due to the inherent overlay error of the e-beam exposure system, the perfect alignment between the gate and source/drain contacts, as shown in Fig. 1(c), was not possible in all fabricated GFETs. This led to the scatter in the measured data because, e.g., smaller underlap (or even unintentional overlap) reduced access resistances and therefore increased ${A}_{{\rm{v}}}$ and ${S}_{21}$, but at the same time reduced ${f}_{{\rm{T}}}$ and ${f}_{{\rm{\max }}}$ due to the increase of parasitic capacitances.

The measured forward transmission coefficient ${S}_{21}$ as a function of $W/L$ is shown in Fig. 3(b). This coefficient reflects the real power amplification as ${|{S}_{21}|}^{2}$ is equal to the transducer power gain of a two-port network. We found that $|{S}_{21}|$ scales with $W/L$ which was expected as it is proportional to ${g}_{{\rm{m}}}$. The narrowest devices (*W*=10 *μ*m) exhibited $|{S}_{21}| < 0$ dB, i.e., power attenuation. The highest measured value was 12.5 dB at 10 MHz for 10 times wider devices. This is also the highest $|{S}_{21}|$ reported in literature for a graphene channel with *W*=100 *μ*m^12^.

**Figure 4:**
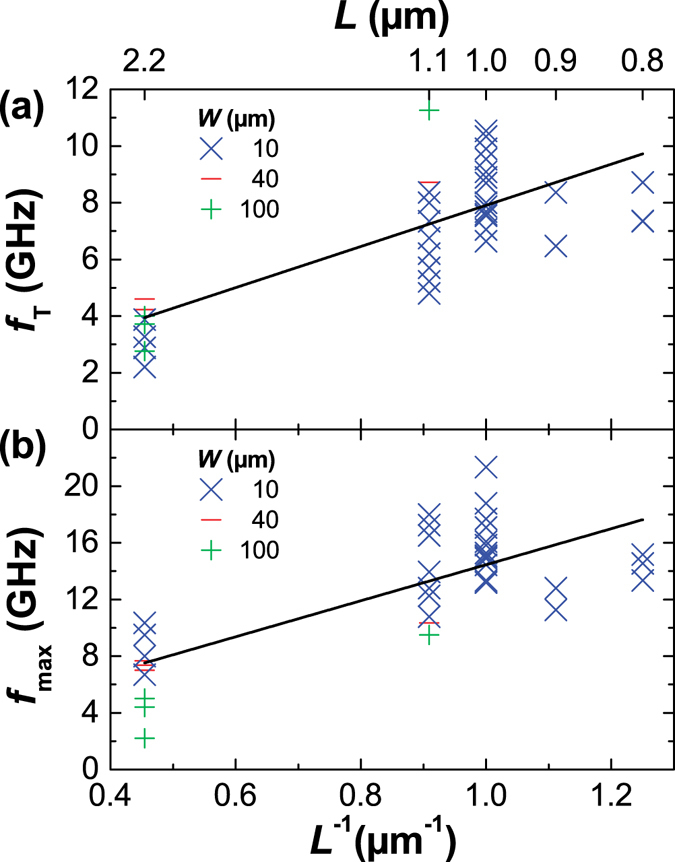
The largest values of the high-frequency transistor response parameters of each fabricated GFET as a function of gate length $L$. (**a**) The cuttof frequency ${f}_{{\rm{T}}}$ and (**b**) maximum oscillation frequency ${f}_{{\rm{\max }}}$. A ${L}^{-1}$ fit is suggested by the black line in both plots.

Figure 4(a) shows the cutoff frequency ${f}_{{\rm{T}}}$ measured on each GFET and plotted as a function of the gate length *L*. The highest measured ${f}_{{\rm{T}}}$ was 10.3 GHz in a device with *W* = 10 *μ*m and *L* = 1 *μ*m. The cutoff frequency is strongly affected by relatively low charge-carrier mobility (*μ* < 1000 cm^2^/(Vs)). Mobilities above 5000 cm^2^/(Vs) have been obtained in CVD graphene deposited on SiO_2_^26, 27^, but the top gate introduces charge traps at the interface with graphene that act as scattering centers degrading transistor performances. The cutoff frequency ${f}_{{\rm{T}}}$ was found to scale approximately with ${L}^{-1}$, as suggested by the black line in Fig. 4(a). However, the scaling trend is not preserved at submicron gate lengths due to the increased influence of contact resistance in shorter devices.

Figure 4(b) shows the highest measured maximum oscillation frequency ${f}_{{\rm{\max }}}$ obtained for each GFET and plotted as a function of the gate length $L$. The highest measured extrinsic ${f}_{{\rm{\max }}}$ was 21.3 GHz in a GFET with *W* = 10 *μ*m and *L* = 1 *μ*m (discussion on the intrinsic ${f}_{{\rm{\max }}}$ can be found in the Supplementary information). The obtained values are comparable to those of GFETs with the same gate length^14, 16, 28^. Even though it has previously been found that ${f}_{{\rm{\max }}}$ does not scale very well with the gate length in GFETs^28^, we observed a gate-length dependence ${L}^{-1}$ down to 0.8 *μ*m, despite noticeable degradation in submicron devices. We obtained larger ${f}_{{\rm{\max }}}$ in narrower devices confirming expected scaling ${f}_{{\rm{\max }}}\propto {R}_{{\rm{g}}}^{-\mathrm{1/2}}\propto {W}^{-\mathrm{1/2}}$, where ${R}_{{\rm{g}}}$ is the gate resistance^28^. Even larger ${f}_{{\rm{\max }}}$ can be obtained if the contact and gate width are adjusted to the actual channel width (i.e., not as in the presented GFETs in which the same metal layout shown in Fig. 1 was used regardless of the channel width). In such devices, the highest measured ${f}_{{\rm{\max }}}$ was 26 GHz with *W* = 10 *μ*m and *L* = 1 *μ*m. However, in general, narrower channels result in smaller gain $|{S}_{21}|$, i.e., there is a tradeoff between $|{S}_{21}|$ and ${f}_{{\rm{\max }}}$.

**Figure 5:**
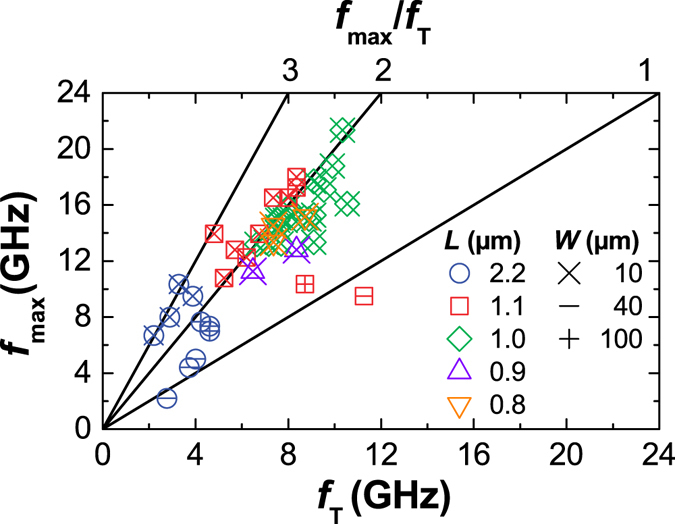
The maximum oscillation frequency ${f}_{{\rm{\max }}}$ as a function of the cutoff frequency ${f}_{{\rm{T}}}$ of each fabricated GFET for different gate lengths $L$ and channel widths $W$. The ratio ${f}_{{\rm{\max }}}/{f}_{{\rm{T}}}$ varies between 1 and 3.

Figure 5 shows the maximum oscillation frequency ${f}_{{\rm{\max }}}$ as a function of the cutoff frequency ${f}_{{\rm{T}}}$. Larger ratio ${f}_{{\rm{\max }}}/{f}_{{\rm{T}}}$ usually indicates better drain current saturation because, to a first approximation, ${f}_{{\rm{\max }}}/{f}_{{\rm{T}}}\propto {g}_{{\rm{d}}}^{-\mathrm{1/2}}$ ^28^. In InP and GaAs high electron mobility transistors, the fastest available technologies, ${f}_{{\rm{\max }}}/{f}_{{\rm{T}}} \sim 2$ ^28^, with the highest measured value of 3.1 in InP transistors exhibiting ${f}_{{\rm{\max }}}=1.2$ THz^29^. In most of the GFETs realized so far, ${f}_{{\rm{\max }}}$ is usually smaller than ${f}_{{\rm{T}}}$ ^13, 14^. A ratio of 1.5 has been obtained in GFETs by reducing the gate resistance through a T-gate structure^15, 16^. The highest ratio reported so far in GFETs has been 3.3 but it has been obtained at ${V}_{{\rm{D}}S}=7$ V^17^. In the present work, we obtained the highest measured ${f}_{{\rm{m}}{\rm{a}}{\rm{x}}}/{f}_{{\rm{T}}}=3.2$, with an average value of 2 for GFETs with $W\mathrm{=10}$ *μ*m. This compares favorably to the state of the art GFETs because all our measurements were performed at $|{V}_{{\rm{DS}}}| < 2$ V. Therefore, an improvement of cutoff frequency by mobility enhancement and contact resistance reduction are expected to increase ${f}_{{\rm{T}}}$ and therefore to increase ${f}_{{\rm{\max }}}$ beyond that of the state of the art GFETs.

## Conclusion

We have demonstrated GFETs with a thin layer of AlO_*x*_ (${t}_{{\rm{ox}}}=4$ nm) gate dielectric. The fabricated GFETs exhibit intrinsic gain ${g}_{{\rm{m}}}/{g}_{{\rm{d}}}\,\mathrm{ > 30}$ dB, forward gain ${S}_{21} \sim 12$ dB, and ratio ${f}_{{\rm{\max }}}\,/{f}_{{\rm{T}}} \sim 3$, at different transistor geometries. The highest ${f}_{{\rm{\max }}}=21.3$ GHz was obtained at a gate length $L=1$ *μ*m indicating adverse influence of contact resistance at shorter gate lengths. However, the critical technological parameters that should be optimized to improve ${f}_{{\rm{\max }}}$ have been identified and they are mostly technological rather than fundamental in nature. The quality of CVD graphene should be enhanced to provide better homogeneity, allowing a more accurate study of the improvement of transistor performance with scaling. Smoother substrates and less defective interfaces should be used to reduce the scattering and increase carrier mobility. Contact resistance should be reduced below 100 Ω*μ*m to simultaneously improve gain and frequency performances. Finally, the gate resistance should be reduced to enhance ${f}_{{\rm{\max }}}$, which can be accomplished through a T-gate structure. Our study has therefore also served to emphasize several remaining challenges of graphene technology which should be overcome to further expand applications of GFETs in electronics.

## Electronic supplementary material

Supplementary Info
