# Supplementary material for: High-Gain Graphene Transistors with a Thin AlOx Top-Gate Oxide
Source: Sci Rep. 2017 May 25;7:2419. doi: 10.1038/s41598-017-02541-2 (PMC5445082; doi:10.1038/s41598-017-02541-2)
Supplement: Supplementary file 1 — Supplementary Info [file 41598_2017_2541_MOESM1_ESM.pdf]

High-Gain Graphene Transistors With a Thin AlO_x Top-Gate Oxide

Erica Guerriero[1], Paolo Pedrinazzi[1], Aida Mansouri[1], Omid Habibpour[2], Michael Winters[2], Niklas Rorsman[2], Ashkan Behnam[3], Enrique A. Carrion[3], Amaia Pesquera[4], Alba Centeno[4], Amaia Zurutuza[4], Eric Pop[5], Herbert Zirath[2], and Roman Sordan[1,*]

[1]L-NESS, Department of Physics, Politecnico di Milano, Polo di Como, Via Anzani 42, 22100 Como, Italy
[2]Department of Microtechnology and Nanoscience, Chalmers University of Technology, Gothenburg, 41296, Sweden
[3]Department of Electrical and Computer Engineering, University of Illinois, Urbana IL 61801, USA
[4]Graphenea, Avenida de Tolosa 76, 20018 Donostia/San Sebastián, Spain
[5]Department of Electrical Engineering, Stanford University, Stanford, CA 94305, USA
[*]roman.sordan@polimi.it

Supplementary Information

Fig. S1 shows the unilateral power gain (U) as a function of frequency (f) of one of the fabricated graphene field-effect transistors (GFETs). As measured, the GFET exhibits maximum oscillation frequency $f_{\mathrm{max}} = 17.7$ GHz. The corresponding intrinsic GFET exhibits $f_{\mathrm{max}} = 41.9$ GHz. If only the contact resistances source/graphene (R_{S}) and drain/graphene (R_{D}) are de-embedded, f_{max} increases to 19.4 GHz. If only the gate resistance (R_{G}) is de-embedded, f_{max} increases to 32.3 GHz. This indicates that T-gate, rather than the conventional gate used in this work, is more suitable for high-frequency operation.

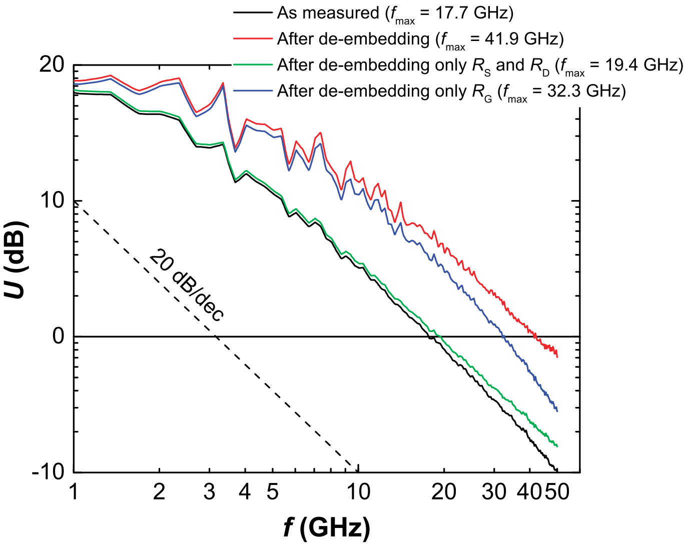

Figure S1. Unilateral power gain measured in a GFET with $W = 10$ μm and $L = 1$ μm.
